# Digitoxin in heart failure with reduced ejection fraction according to atrial fibrillation status: the DIGIT-HF trial

**DOI:** 10.1093/eurheartj/ehag379

**Published:** 2026-05-10

**Authors:** Udo Bavendiek, Nele Henrike Thomas, Lars S Maier, Dominik Berliner, Peter M Becher, Johannes Schwab, Andreas Rieth, Thomas Gaspar, Xiaofei Liu, Sven Schallhorn, Samira Soltani, Lea Haebel, Marija Zdravkovic, Martin Hülsmann, Anika Seltmann, Paulus Kirchhof, Heiko von der Leyen, Christian Veltmann, Stefan Störk, Michael Böhm, Armin Koch, Anika Großhennig, Johann Bauersachs

**Affiliations:** Department of Cardiology and Angiology, Hannover Medical School, Carl-Neuberg-Straße 1, Hannover 30625, Germany; Institute for Biostatistics, Hannover Medical School, Hannover, Germany; Department for Internal Medicine II, University Hospital Regensburg, Regensburg, Germany; Department of Cardiology and Angiology, Hannover Medical School, Carl-Neuberg-Straße 1, Hannover 30625, Germany; Department of Cardiology, University Heart and Vascular Center Hamburg, Hamburg, Germany; Department of Cardiology, Paracelsus Medical University, Nuremberg, Germany; MVZ Kardiologie, Klinikum Neumarkt, Neumarkt, Germany; Department of Cardiology, Kerckhoff-Klinik, Bad Nauheim, Germany; Department of Internal and Cardiovascular Medicine, Herzzentrum Dresden, University Clinic, Dresden, Technische Universität Dresden, Dresden, Germany; Department of Cardiology, Angiology and Internal Intensive Care Medicine, Medical Clinic 2, Städtisches Klinikum Dresden, Dresden, Germany; Institute for Biostatistics, Hannover Medical School, Hannover, Germany; Department of Cardiology and Angiology, Hannover Medical School, Carl-Neuberg-Straße 1, Hannover 30625, Germany; Department of Cardiology and Angiology, Hannover Medical School, Carl-Neuberg-Straße 1, Hannover 30625, Germany; Department of Cardiology and Angiology, Hannover Medical School, Carl-Neuberg-Straße 1, Hannover 30625, Germany; Clinic for Internal Medicine, University Clinical Hospital Center Bezanijska Kosa, Faculty of Medicine, University of Belgrade, Belgrade, Serbia; Universitätsklinik für Innere Medizin II, Abteilung Kardiologie, Medizinische Universität Wien, Wien, Austria; Center for Clinical Trials, Hannover Medical School, Hannover, Germany; Department of Cardiology, University Heart and Vascular Center Hamburg, Hamburg, Germany; German Centre for Cardiovascular Research, Partner Site North, Hamburg, Germany; Institute of Cardiovascular Sciences, University of Birmingham, Birmingham, UK; Orgenesis, Inc, Germantown, USA; Hannover Medical School, Germany; Department of Cardiology and Angiology, Hannover Medical School, Carl-Neuberg-Straße 1, Hannover 30625, Germany; Center for Electrophysiology Bremen, Bremen, Germany; Department Clinical Research & Epidemiology, Comprehensive Heart Failure Center Würzburg, and Department Internal Medicine I, University Hospital Würzburg, Würzburg, Germany; HOMICAREM (HOMburg Institute for CArdioREnalMetabolic Medicine), Medical Faculty, Campus Universitätsklinikum des Saarlandes, Saarland University, Homburg/Saar, Germany; Institute for Biostatistics, Hannover Medical School, Hannover, Germany; Institute for Biostatistics, Hannover Medical School, Hannover, Germany; Department of Cardiology and Angiology, Hannover Medical School, Carl-Neuberg-Straße 1, Hannover 30625, Germany

## Background

*Post hoc*-analyses of various clinical trials and registries describe heterogenous effects of cardiac glycosides on morbidity and mortality in patients with heart failure (HF) with and without atrial fibrillation (AF).^1,2^ However, prospective data on the impact of cardiac glycosides on hard clinical outcomes in patients with concomitant HFrEF and AF are lacking. Moreover, in the presence of AF, several therapies recommended for HF with reduced ejection fraction (HFrEF) are either not indicated (such as ivabradine) or less effective, including beta-blockers and cardiac resynchronization therapy.^3,4^ The DIGIT-HF trial recently demonstrated that low-dose digitoxin, added to well-implemented guideline-recommended HF therapy (GRMT), reduced the primary composite outcome of first hospitalization for worsening HF (HFH) or all-cause death (ACD) in patients with HFrEF.^5^ Unlike the earlier DIG trial of digoxin,^6^ DIGIT-HF enrolled patients both with and without AF. In this *post hoc* analysis, we therefore investigated whether the efficacy of digitoxin differed according to the diagnosis of AF at baseline.

## Methods

DIGIT-HF was a randomized, double-blind, placebo-controlled multicentre trial evaluating the efficacy and safety of digitoxin in patients with symptomatic HFrEF (LVEF ≤30% and NYHA class II, or LVEF ≤40% and NYHA class ≥ III). Patients were randomized to receive digitoxin or placebo once daily in addition to GRMT. Digitoxin was initiated at 0.07 mg daily and titrated in a blinded fashion to 0.05 mg or 0.1 mg according to a prespecified protocol to achieve low serum concentrations of 8–18 ng/mL. The trial’s design, baseline characteristics, and primary results have been previously published.^5,7,8^ In this *post hoc* analysis, we compared the primary composite outcome (time to HFH or ACD), and its individual components, between patients with and without the diagnosis of AF at baseline. Baseline characteristics were summarized using median (interquartile range [IQR]) or counts (percentage) and compared using two-sided Wilcoxon rank sum tests or Chi-Square tests, as appropriate. Cox regression models were employed to evaluate time-to-event outcomes between patients with and without AF adjusting for treatment assignment, study site, sex, and NYHA class; additional adjustments were made for baseline variables that differed between patients with and without AF. To assess the effect of digitoxin according to AF status, Cox regression models adjusting for study site, sex, and NYHA class were utilized, again additional adjustments were made for baseline variables that varied between digitoxin and placebo patients with and without AF. In addition, the interaction between AF status and treatment was assessed in the primary analysis model. Analyses followed the intention-to-treat (ITT) principle and were conducted using SAS software (version 9.4; SAS Institute, Cary, USA). Hazard ratios (HRs), respective 95% confidence intervals (CIs), and descriptive *P*-values are reported. *P*-values <.05 were considered statistically significant.

## Results

Of the 1212 patients included in the intention-to-treat analysis, 330 (27%) had a diagnosis of AF at baseline. Compared with those without AF, patients with AF were older and had a longer history of HF, had more severe HF symptoms, higher heart rates, lower blood pressure, and worse renal function; further baseline differences are displayed in *Figure 1A*. The composite endpoint of HFH or ACD, and its individual components, occurred more frequently in patients with AF than in those without (*Figure 1B*). After adjustment for prognostic variables that differed by AF status, however, HRs and CIs were similar across the two groups (*Figure 1B*). Within each rhythm stratum, the randomized treatment arms were largely balanced. There were a few minor imbalances; amongst patients with AF, digitoxin-treated patients had lower heart rates (75 ± 13 vs 79 ± 14 b.p.m, *P* = .02), lower frequency of chronic obstructive lung disease (43 vs 51%, *P* = .022), and higher frequency of diabetes (31 vs 43%, *P* = .023); amongst patients without AF, digitoxin-treated patients had a higher frequency of hyperlipidemia (63 vs 57%, *P* = .047) and ICD implants (72 vs 63%, *P* = .004). Sensitivity analysis accounted for these differences.

**Figure 1:**
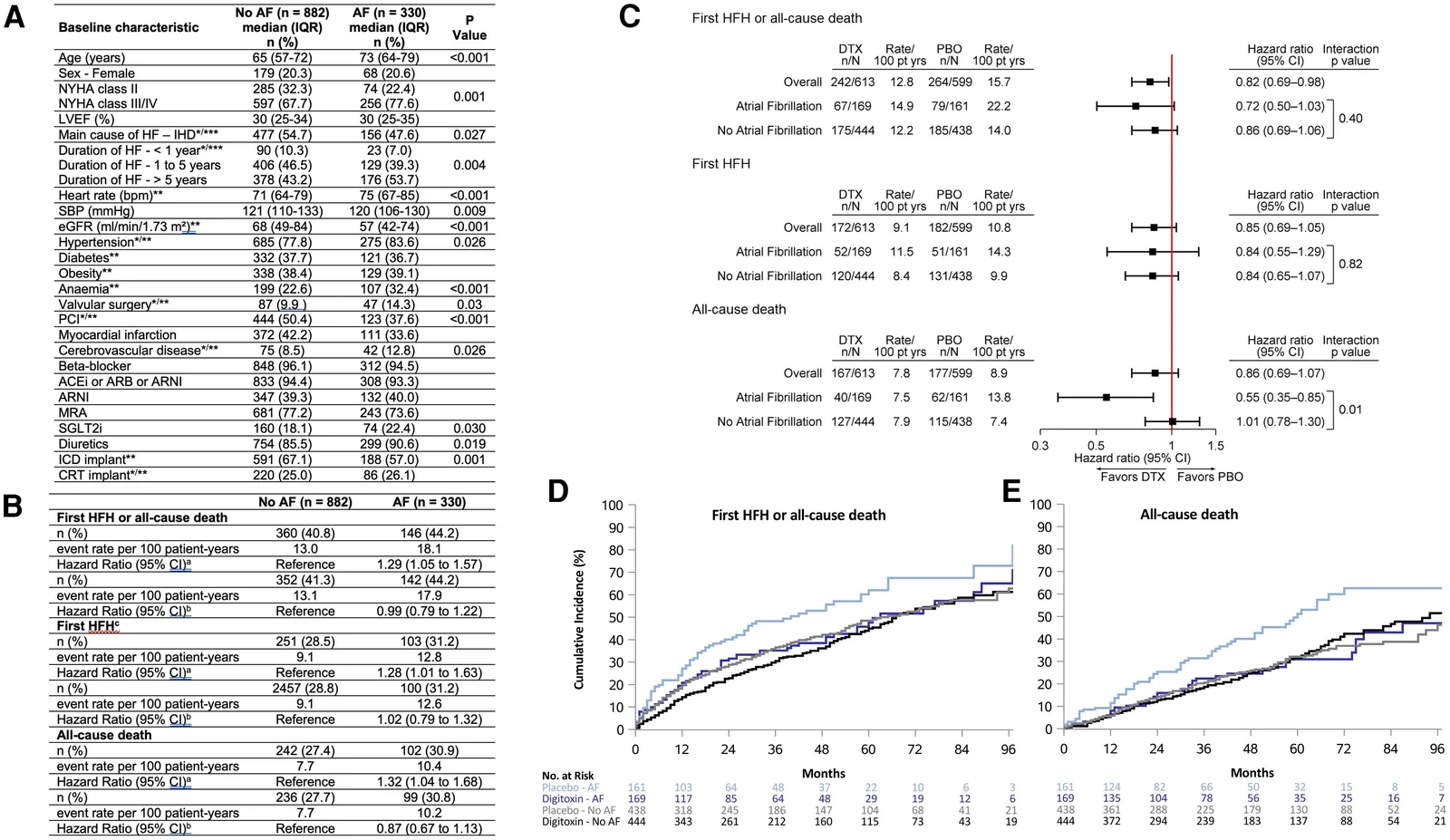
A: Key baseline characteristics of patients according to AF status at baseline; B: Time to first event according to AF status at baseline; C: Effect of digitoxin on outcomes according to AF status at baseline; D/E: Cumulative incidence of first heart failure hospitalization (HFH) or all-cause death (*D*) and all-cause death (*E*) in treatment groups according to AF status at baseline. AF, atrial fibrillation; IQR, interquartile range; NYHA, New York Heart Association; LVEF, left ventricular ejection fraction; SBP, systolic blood pressure; eGFR, estimated glomerular filtration rate; PCI, percutaneous coronary intervention; ACEi, angiotensin-converting enzyme inhibitor; ARB, angiotensin-receptor blocker; ARNI, angiotensin receptor-neprilysin inhibitor; MRA, mineralocorticoid receptor antagonist; SGLT2i, sodium-glucose cotransporter 2 inhibitor; ICD, implantable cardioverter-defibrillator; CRT, cardiac-resynchronization therapy. *One to three missing values for indicated variables for patients with AF. **One to three missing values for indicated variables for patients with no AF. ***Ten missing values for main cause of HF, and eight missing values for duration of HF in patients with no AF. ^a^Cox proportional hazards regression models adjusted in line with the primary analysis for AF, i.e. assigned treatment group, study site, sex, and NYHA functional class. ^b^Cox proportional hazards regression models as described above and additionally adjusted for potentially relevant baseline differences, i.e. age, heart rate, systolic blood pressure, eGFR, main cause of heart failure, duration of heart failure, hypertension, anaemia, valvular surgery, percutaneous coronary intervention, myocardial infarction, cerebrovascular disease. If missing values occurred in one of the covariables, the respective patient was deleted from the analysis. ^c^Data for patients who died without being hospitalized were censored at the date of death

In the overall cohort, digitoxin significantly reduced the primary endpoint—with reductions of similar magnitude observed for HFH and ACD (*Figure 1C*). This benefit was evident in both AF and non-AF patients (*Figure 1C & D*). The absolute risk reduction for the composite endpoint was 9.5% in the AF group vs 2.8% in the non-AF group, yielding numbers needed to treat over the 36-month follow-up of 11 and 36, respectively; yet, there was no significant interaction between AF status and treatment effect (*P*_interaction_ = .4). For ACD alone, the reduction appeared to be more pronounced in patients with AF than in those without (*Figure 1C & E*) with absolute risk reductions of 14.8% vs 2.3%, and numbers needed to treat of 7 vs 44, respectively (*P*_interaction_ = .01), whereas reductions in first HFH were consistent regardless of AF status (*Figure 1C*). Adjusting for the aforementioned baseline imbalances did not materially alter these results.

## Discussion

DIGIT-HF currently represents the only prospective, randomized, and placebo-controlled clinical trial providing evidence that a cardiac glycoside can improve hard outcomes in patients with concomitant HFrEF and AF.^5^ Consistent with previous reports,^9^ the subset of participants with AF constituted a clinically more advanced cohort than those without, with a heavier burden of comorbidities and more severe symptoms. Nonetheless, digitoxin reduced the composite of first HFH or ACD irrespective of AF status. Although the formal interaction between AF and treatment did not reach significance, the larger absolute risk reductions and lower numbers needed to treat in the AF subgroup—driven predominantly by reduced mortality—suggest that digitoxin may confer particular benefit in this high-risk population. Importantly, these benefits were achieved on top of contemporary GRMT and without evidence of harm. Further evidence regarding the efficacy of cardiac glycosides in HFrEF and AF is expected from the DECISION trial, which is evaluating low-dose digoxin in symptomatic HF patients (LVEF ≤50%).^10^

In summary, our findings suggest that digitoxin appears to be effective and safe in patients with HFrEF, including those with AF.

## Declarations

### Data Availability

The data that support the findings of this paper are available from the corresponding author upon reasonable request.

### Pre-registered Clinical Trial Number

Clinical Trial Registration: EudraCT (2013–005326-38).

## DIGIT-HF study group

### Investigators by country

#### Germany

Katharina Marx-Schütt, Nikolaus Marx, Uniklinik RWTH Aachen

Andreas Rieth, Veselin Mitrovic, Kerckhoff-Klinik GmbH Bad Nauheim

Alessandro Cuneo, Sergiu Hicea, Klinikum Westmünsterland Ahaus

Andreas Ritze, Johannes Haas, CIMS Studienzentrum Bamberg GmbH Bamberg

Frank Edelmann, Tobias Trippel, Charité Campus Virchow Klinikum Berlin

Sebastian Winkler, Mirko Seidel, Unfallkrankenhaus Berlin

Bettina Heidecker, Arash Haghikia, Charité Universitätsmedizin Berlin-CBF Berlin

Monika Ernst, Christoph Hanefeld, Katholisches Klinikum Bochum gGmbH, St. Elisabeth-Hospital Bochum

Kristina Becker, Andreas Mügge, Katholisches Klinikum Bochum gGmbH, Sankt Josef-Hospital Bochum

Georg Nickenig, Can Öztürk, Universitätsklinikum Bonn

Rainer Hambrecht, Harm Wienbergen, Klinikum Links der Weser Herzzentrum Bremen

Johannes Brachmann, Steffen Schnupp, Klinikum Coburg

Ralph Oeckinghaus, Timo Aschenbrenner, Klinikum Lippe Detmold

Thomas Paul Gaspar, Stefan Ulbrich, Herzzentrum Dresden GmbH-Universitätsklinikum Dresden

Ruth Strasser, Stafanie Katzke, Herzzentrum Dresden GmbH, Universitätsklinik an der Technischen Universität Dresden

Christian Meyer, Bahram Wafaisade, Evangelisches Krankenhaus Düsseldorf

Daniel Scheiber, Malte Kelm, Universitätsklinikum Düsseldorf

Till Neumann, Hagen Kälsch, Universitätsklinikum Essen

Constantin von zu Mühlen, Sebastian Grundmann, Universitätsklinikum Freiburg

Kristian Hellenkamp, Tim Seidler, Universitätsmedizin Göttingen

Alexander Vogt, Jochen Dutzmann, Universitätsklinikum Halle

Herbert Nägele, Philipp Peitsmeyer, Albertínen-Krankenhaus Hamburg

Moritz Becher, Nina Fluschnik, Universitätsklinikum Hamburg-Eppendorf Hamburg

Udo Bavendiek, Johann Bauersachs, Medizinische Hochschule Hannover

Lutz Frankenstein, Tobias Täger, Universitätsklinikum Heidelberg

Michael Böhm, Ingrid Kindermann, Universitätsklinikum des Saarlandes Homburg/Saar

Christian Schulze, Julian Georg Westphal, Universitätsklinikum Jena

Roman Pfister, Stephan Rosenkranz, Herzzentrum Uniklinik Köln

Marcus Sandri, Holger Thiele, Leipzig Heart Institute GmbH Leipzig

Rolf Wachter, Michael Metze, Universität Leipzig

Tobias Graf, Christian Reil, Universitätsklinikum Schleswig-Holstein Lübeck

Rüdiger Braun-Dullaeus, Alexander Schmeißer, Otto-von-Guericke-Universität Magdeburg Universitätsklinikum Magdeburg A. ö. R. Magdeburg

Philip Wenzel, Tommaso Gori, Universitätsmedizin Johannes-Gutenberg-Universität Mainz

Michael Behnes, Ibrahim Akin, Universitätsklinikum Mannheim

Bernhard Schieffer, Wolfram Grimm, Universitätsklinikum Gießen und Marburg GmbH Standort Marburg

Jens Taggeselle, Antje Stumpp, Internistische Praxis Dr. Taggeselle Markkleberg

Roland Prondzinsky, Susanne Rode, Carl von Basedow Klinikum Saalekreis GmbH Merseburg

Norbert Schön, Brigitte Schön, Kardiologisch-Angiologische Schwerpunktpraxis Mühldorf am Inn

Stefan Kääb, Stefan Brunner, Universitätsklinikum München Campus Innenstadt München

Johannes Schwab, Matthias Pauschinger, Klinikum Nürnberg Süd

Andreas Götte, Sibylle Brandner, St. Vinzenz-Krankenhaus GmbH Paderborn

Uwe Gremmler, Birgit Gerecke, Kardiologisches Zentrum Peine MVZ Peine

Lars Maier, Bernhard Unsöld, Universitätsklinikum Regensburg

Markus Schwefer, Stefan Hettwer, Elblandklinikum Riesa

Stefan Rausch, Kyrill Rogacev, MVZ Schwerin West GmbH Schwerin

Sebastian Philipp, Torsten Lauf, Elbe Kliniken Stade-Buxtehude GmbH Stade

Stefan Störk, Caroline Morbach, Universitätsklinikum Würzburg

#### Austria

Martin Hülsmann, Suriya Prausmüller, Henrike Arfsten, Medizinische Universität Wien

Johann Auer, A. ö. Krankenhaus Sankt Josef Braunau GmbH Braunau

#### Serbia

Marija Zdravkovic, Klinicko bolnicki centar Bezanijska Kosa Belgrade

Dejan Spiroski, Institut za rehabilitaciju Belgrade

Dragana Kosevic, Institut Za Kardiovaskularne Bloesti "Dedinje" Belgrade

Natasa Markovic Nikolic, Klinicko Bolnicki Centar "Zvezdara"- Beograd Belgrade

Svetlana Apostolovic, Univerzitetski Klinicki Centar Nis Nis

Marina Deljanin Ilic, Institut Za Lecenje I Rehabilitciju “Niska Banja” Nis Niska Banja

### Committees and Study Personnel

#### Trial Steering Committee (TSC)

Udo Bavendiek (Head of Study), Medizinische Hochschule Hannover, Germany

Johann Bauersachs (Head of Study), Medizinische Hochschule Hannover, Germany

Armin Koch (Statistician of TSC), Medizinische Hochschule Hannover, Germany

Christian Veltmann (Medical Expert), Elektrophysiologie Bremen, Germany

Michael Böhm (Medical Expert), Universitätsklinikum Homburg/Saar, Germany

Heiko von der Leyen (Medical Expert), Medizinische Hochschule Hannover, Germany

Stefan Störk (Medical Expert), Universitätsklinikum Würzburg, Germany

#### Data Monitoring Committee (DMC)

Stefan Anker (Head of DMC), Charite Universitätsmedizin Berlin, Germany

Hans J. Trampisch (Statistician of DMC), Ruhr-Universität Bochum, Germany

Paul Mohacsi (Medical Expert), HerzGefäßZentrum im Park Hirslanden, Switzerland

Gerhard Pölzl, Medizinische Universität Innsbruck, Austria

#### Clinical Event Adjudication Committee (CEAC)

Ulrich Tebbe, Institut für Klinische Forschung Göttingen, Germany

Markus Haas, Theresienkrankenhaus Mannheim, Germany

Stephan von Haehling, Universitätsmedizin Göttingen, Germany

#### National Lead Investigators

Udo Bavendiek (Head of study), Medizinische Hochschule Hannover, Germany

Marija Zdravkovic, Klinicko bolnicki centar Bezanijska Kosa Belgrade, Serbia

Martin Hülsmann, Medizinische Universität Wien, Austria

#### Principal Investigators and Sponsor

Udo Bavendiek (Head of Study), Medizinische Hochschule Hannover, Germany

Johann Bauersachs (Head of Study), Medizinische Hochschule Hannover, Germany

Armin Koch (Statistician of TSC), Medizinische Hochschule Hannover, Germany

Christoph Schindler (Sponsor Representative), Medizinische Hochschule Hannover, Germany

Dirk O. Stichtenoth (Pharmacovigilance), Medizinische Hochschule Hannover, Germany
